# Activity Pattern Analysis Indicates Increased but Balanced Systemic Coagulation Activity in Response to Surgical Trauma

**DOI:** 10.1055/s-0038-1673390

**Published:** 2018-10-01

**Authors:** Max Julian Friedrich, Jan Schmolders, Yorck Rommelspacher, Andreas Strauss, Heiko Rühl, Günter Mayer, Johannes Oldenburg, Dieter Christian Wirtz, Jens Müller, Bernd Pötzsch

**Affiliations:** 1 Department of Orthopedics and Trauma Surgery, University Hospital Bonn, Bonn, Germany; 2 Institute of Experimental Haematology and Transfusion Medicine, University Hospital Bonn, Bonn, Germany; 3 Life and Medical Sciences Institute (LIMES), University of Bonn, Bonn, Germany

**Keywords:** coagulation, surgical hemostasis, DNA aptamers, biomarkers, enzyme activity

## Abstract

In the nonbleeding patient, constant low-level activation of coagulation enables a quick procoagulant response upon an injury. Conversely, local activation of coagulation might influence the systemic activity level of coagulation. To characterize this interaction in more detail, activity pattern analysis was performed in patients undergoing elective surgeries. Blood samples were taken before, during, and 24 hours after surgery from 35 patients undergoing elective minor ( *n* = 18) and major ( *n* = 17) orthopaedic surgeries. Plasma levels of thrombin and activated protein C (APC) were measured using oligonucleotide-based enzyme capture assays, while those of prothrombin fragment 1.2, thrombin–antithrombin-complexes, and D-dimer were measured using commercially available enzyme-linked immunosorbent assays. In vitro thrombin generation kinetics were recorded using calibrated automated thrombography. Results showed that median plasma levels of up to 20 pM thrombin and of up to 12 pM APC were reached during surgery. D-dimer levels started to increase at the end of surgery and remained increased 24 hours after surgery, while all other parameters returned to baseline. Peak levels showed no significant differences between minor and major surgeries and were not influenced by the activity state at baseline. In vitro thrombin generation kinetics remained unchanged during surgery. In summary, simultaneous monitoring of the procoagulant and anticoagulant pathways of coagulation demonstrates that surgical trauma is associated with increased systemic activities of both pathways. Activity pattern analysis might be helpful to identify patients at an increased risk for thrombosis due to an imbalance between surgery-related thrombin formation and the subsequent anticoagulant response.

## Introduction

Coagulation is a dynamic and temporal-spatial controlled process that becomes activated after vessel wall injury by complex formation between activated factor VII (FVIIa) and tissue factor (TF). 1 The resulting extrinsic activation complex activates factor X that subsequently catalyzes the formation of subnanomolar amounts of thrombin. 2 This initial thrombin recruits and activates platelets and augments further thrombin formation through a series of acceleration steps resulting in peak thrombin levels of approximately 800 nM as measured through in vitro monitoring of TF-induced coagulation activation. 3 Since formation of a stable clot is achieved at thrombin concentrations between 10 and 20 nM, it is concluded that 96% of thrombin is generated after the wound-sealing clot has been formed. Part of this thrombin is released into the flowing blood, since thrombin, unlike other activated coagulation factors, contains no phospholipid-binding sites. A fraction of this blood-born thrombin is rapidly inactivated by stoichiometric inhibitors such as antithrombin (AT), heparin cofactor II, and α-2-macroglobin. 4 The resulting thrombin–AT (TAT) complex circulates in blood with a half-life of 44 minutes. 5 Another fraction of the blood-born thrombin binds to thrombomodulin (TM) on the surface of endothelial cells where it becomes an anticoagulant through conversion of protein C (PC) into the active enzyme activated protein C (APC). 6 Taken together, these thrombin-inhibiting mechanisms limit the half-life of thrombin in the circulating blood to about 60 seconds. 7 Despite this short half-life, the results of numerical simulation of thrombin profiles have predicted thrombin blood levels reaching low nanomolar concentrations downstream a wounded area. 8 9 Hence, localized coagulation activation should induce a systemic coagulation response.

Recently, we measured systemic thrombin levels in the picomolar range in surgical patients in a small pilot study. 10 To make these low thrombin concentrations measurable, a highly sensitive oligonucleotide (aptamer)-based enzyme capture assay (OECA) was combined with a blood sampling technique that protects thrombin from early inactivation by endogenous inhibitors through addition of the reversible thrombin inhibitor argatroban to the blood sampling buffer. 10 The thrombin–OECA captures thrombin–argatroban complexes using a bivalent thrombin-specific aptamer that binds to both exosite motifs of the enzyme. After immobilization, argatroban is washed out and thrombin quantified through hydrolysis rates of a fluorogenic peptide substrate. Using this approach, plasma levels of free thrombin have become measurable with lower limits of detection and quantification of 0.46 and 1.06 pM, respectively.

Although the data obtained in a small series of patients undergoing hip- and knee-replacement surgeries using this approach have given first evidence for the presence of low levels of circulating thrombin, the influence of spatial coagulation activation on the systemic coagulation reactions has not been studied in detail. To quantify the systemic coagulation response and to identify factors that influence the activity levels, we performed activity pattern analysis in patients undergoing a broad range of elective orthopaedic surgeries. Besides thrombin, plasma levels of the prothrombin activation fragment 1.2 (F1.2) and TAT were measured to additionally assess the total amount of thrombin formed and that of AT-inactivated thrombin, respectively. Plasma levels of APC were quantified as a measure for thrombin-dependent activation of the protein C pathway. 11 Plasma levels of D-dimer were quantified as a measure for the formation of cross-linked fibrin and subsequent degradation by plasmin. In addition, results of in vitro thrombin generation kinetics were compared with in vivo thrombin generation kinetics to study if changes in the systemic coagulation activities influence in vitro thrombin formation kinetics.

## Patients, Materials, and Methods

### Study Design

Patients scheduled for elective orthopaedic surgery and who have given informed consent for study participation were eligible for the study. According to the guidelines for perioperative care published by the American College of Cardiology (ACA) and the American Heart Association (AHA), the surgeries were graded into minor and major surgeries depending on magnitude, type, duration, blood loss, and transfusion requirements. 12 For prevention of thromboembolic events, all patients received enoxaparin once daily at a dosage of 40 mg subcutaneously starting 4 to 8 hours postoperatively. The study was approved by the local ethics committee of the University of Bonn (#001/13).

### Blood Sampling and Processing

Blood samples were taken 2 hours before surgery and 24 hours after surgery by puncturing an antecubital vein using a 21-gauge winged blood collection set (Sarstedt, Nümbrecht, Germany). During surgery, blood was taken from a vein of the lower arm via a 21-gauge indwelling venous cannula (B. Braun, Melsungen, Germany) maintained patent by a continuous infusion of physiological sodium chloride solution. During surgery, blood samples were taken at incision of the skin (incision), at the middle of the surgery, and at final suture of the skin. The middle of the surgeries was defined as removal of the herniated intervertebral disc for nucleotomies, as implantation of the acetabular component for total hip arthroplasties, or by definition of the surgeon intraoperatively for all other types of surgeries. For routine coagulation analyses, blood was collected into 3-mL trisodium citrate tubes (10.6 mM final concentration). For thrombin–OECA or APC–OECA testing, the citrate tubes were prefilled with argatroban (final concentration 100 µmol/L) or aprotinin and r-hirudin (final concentrations 10 µmol/L and 15 µg/mL). Filled blood tubes were stored and transported at room temperature or in crushed ice (APC tubes) and centrifuged within 4 hours at 2,500 × g for 15 minutes, and plasma samples were stored at –40°C until assayed.

### Laboratory Analysis

F1.2 and TAT were measured using Enzygnost F1.2 (monoclonal) and Enzygnost TAT micro enzyme-linked immunosorbent assay kits (Siemens Healthcare Diagnostics, Marburg, Germany). The fibrin fragment D-dimer was measured using the Innovance D-dimer kit and a BCS XP coagulation analyzer (both Siemens Healthcare Diagnostics). All analyses were performed according to the manufacturer's instructions. Levels of thrombin and APC were measured using the thrombin– and APC–OECA as previously described. 10 11 Thrombin generation kinetics were measured using calibrated automated thrombography (CAT) (Thrombinoscope BV, Maastricht, the Netherlands) using standard reagents (Stago, Düsseldorf, Germany) and a Fluoroskan Ascent FL plate reader (Thermo Scientific) as described elsewhere. 13 Reference ranges for all parameters were previously established by analyzing plasma samples of healthy individuals. Normal plasma levels of thrombin and APC were found below the lower limit of quantification (LLOQ) of the assays. 10 11 Thus, the LLOQ was defined as the upper limit of the reference range of these parameters.

### Statistical Analysis

The Wilcoxon signed-rank test with continuity correction (paired) was applied to assess the statistical difference between plasma levels measured at the different time points. The analysis was done online using R-based software as available on the wessa.net web server (Wessa, P. [2018], Free Statistics Software, Office for Research Development and Education, version 1.2.1, URL https://www.wessa.net/ ).

For the assessment of statistical differences between the percentages of values found within or outside the reference ranges, 2 × 2 contingency tables and the Fisher exact probability test were applied. Calculations were done online using the QuickCalcs module on the graphpad.com homepage ( http://graphpad.com/quickcalcs/contingency1.cfm ).

## Results

### Study Patients

Thirty-five patients (14 females) with a mean age of 50.5 ± 17.4 years (mean body mass index, 26.4 ± 2.6) were included in the study. At baseline, all patients showed the following key coagulation parameters within the respective normal ranges: platelet count, prothrombin time, activated partial prothrombin time, factor II activity, factor VII activity, protein C activity and AT activity. The surgical procedures included 13 nucleotomies, 4 total hip arthroplasties, 8 arthroscopic interventions, 5 osteosynthesis procedures, and 5 implant removals. Surgeries were categorized as minor ( *n* = 18) or major ( *n* = 17) according to ACH/AHA criteria. Mean interventions times (mean ± SD, 170 ± 95 vs. 60 ± 33 minutes) and mean blood loss (294 ± 351 vs. 17 ± 37 mL) were significantly higher in the major surgery group (for details, see Supplementary Tables 1A and 1B ). Five patients of the major surgery group required transfusion of packed red blood cells (transfusion trigger: Hb < 80 g/L), whereas none of the patients in the minor surgery group required blood support.

None of the patients had a bleeding history indicative of an inherited or acquired hemostasis disorder or a history of venous thromboembolism. Results of routine coagulation analysis were within the respective reference ranges (data not shown).

### In Vitro Thrombin Generation Kinetics Are Not Influenced by Surgical Trauma

**Fig. 1:**
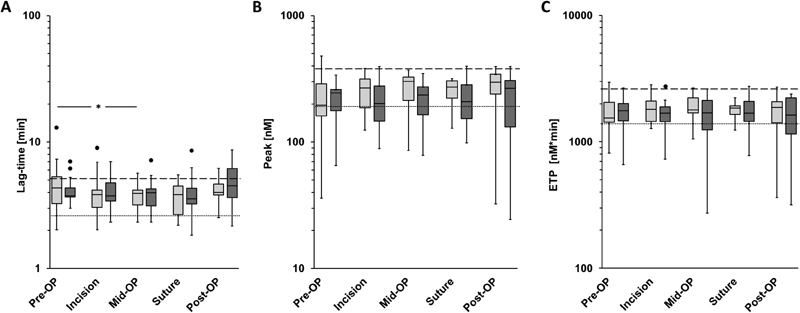
In vitro thrombin generation kinetics in patients undergoing minor ( *light gray boxes* ) and major ( *dark gray boxes* ) orthopaedic surgeries. Changes in ( **A** ) lag time, ( **B** ) peak thrombin generation, and ( **C** ) endogenous thrombin potential (ETP) were measured at the indicated time points using CAT as outlined in the text. Data are expressed as box and whiskers plots with median, minimal and maximal values, interquartile ranges, and outliers. The *dashed* and *dotted horizontal lines* represent the established upper and lower reference limit. Significant differences ( *p* < 0.05) between blood collecting time points are indicated by a line dragged between the groups (-*-).

To study whether surgery-induced activation of coagulation changes in vitro thrombin generation kinetics, we measured the lag time, peak thrombin, and endogenous thrombin potential values after TF-induced (5 pM final concentration) activation of coagulation by CAT. The minor surgery group showed a moderate statistically significant decrease of the median lag time (4.33 vs. 3.93 minutes, *p* = 0.019) at the middle of the surgery compared with baseline, while significant changes to baseline were not observed at all other time points ( Fig. 1 ). The major surgery group showed no significant changes to baseline for all three parameters at any time point.

### Plasma Levels of Thrombin and APC Substantially Increase during Surgery

**Fig. 2:**
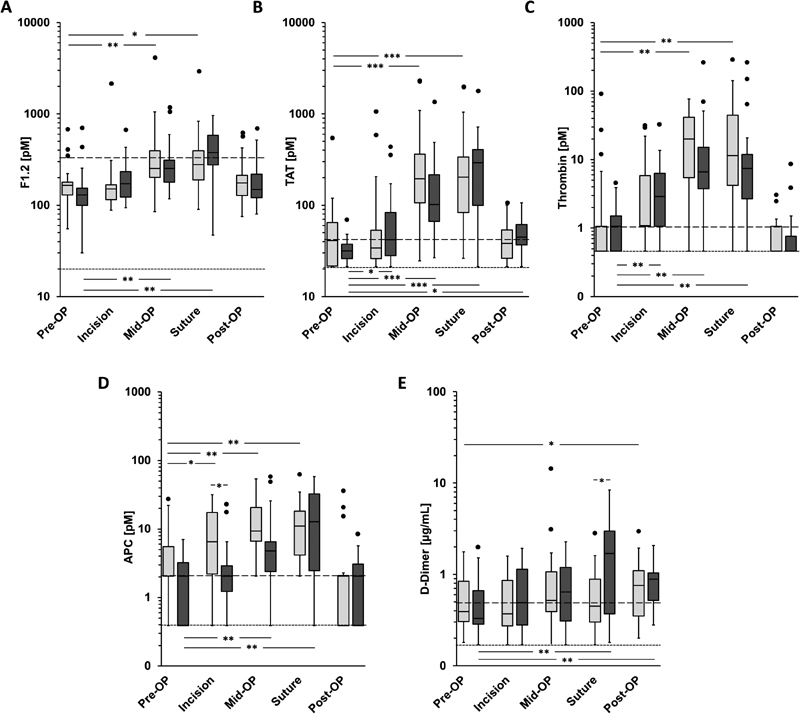
Changes of in vivo coagulation biomarkers during orthopaedic surgeries. Plasma levels of the ( **A** ) prothrombin fragment (F1.2), ( **B** ) thrombin–antithrombin complexes (TAT), ( **C** ) thrombin, ( **D** ) activated protein C (APC), and ( **E** ) D-dimer were measured at the indicated time points in patients undergoing minor ( *light gray boxes* ) and major ( *dark gray boxes* ) orthopaedic surgeries. Data are expressed as box and whiskers plots with median, minimal and maximal values, interquartile ranges, and outliers. The *long* - and *short-dashed horizontal lines* represent the established upper limit of the normal ranges and the LLOQ or LOD (thrombin, APC) of the assays. Significant differences * *p* < 0.05, ** *p* < 0.01, *** *p* < 0.001.

Plasma levels of F1.2 showed a significant increase to baseline at the middle and end of the surgeries and returned to baseline 24 hours after end of surgery ( Fig. 2A ). The increase was comparable in patients undergoing minor and major surgeries. TAT levels increased after start of surgery and reached median plasma levels of 148 and 245 pM at the middle and at end of surgery, respectively ( Fig. 2B ). There was no significant difference between both patient groups at any time point. Mean thrombin levels increased from a median of 0.46 pM (limit of detection [LOD]) at baseline to 1.09 pM at incision and reached median values of 20.05 and of 11.41 pM at the middle and end of major surgeries, respectively ( Fig. 2C ). A nearly identical dynamic of thrombin formation kinetics was measured in minor surgeries with the exception that the increase at incision was not statistically significant. Thrombin levels returned to baseline 24 hours after surgery in both patient groups.

Plasma levels of APC also increased significantly during the surgical interventions in both groups reaching median values of 6.62 and 12.76 pM at the middle and end of the surgeries, respectively. Twenty-four hours after surgery, APC values returned to baseline. With the exception of the time point at incision, there was no statistical difference between plasma APC levels in both patient groups ( Fig. 2D ).

Plasma levels of D-dimer showed no increase to baseline at incision and at the middle of the surgeries in both groups ( Fig. 2E ), but significantly increased at the end of surgery in the major surgery group. Twenty-four hours after end of the surgery, D-dimer levels significantly increased relative to baseline in both groups. There was no statistically significant difference between both groups.

Levels of the different parameters in all analyzed samples are shown in Supplementary Table 2 .

### Color-Coded Visualization of Surgery-Induced Changes in Systemic Coagulation Activity

**Fig. 3:**
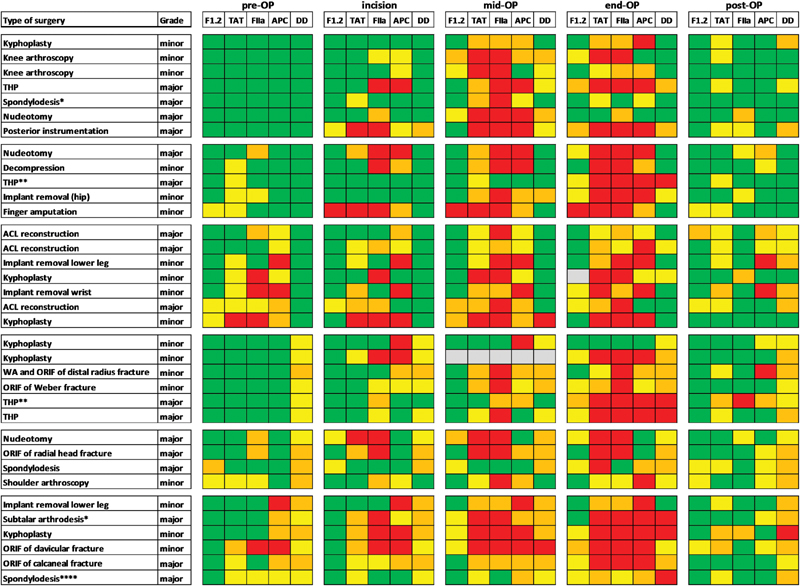
Digital dynamics of surgery-induced changes in activity patterns. The changes in plasma levels of prothrombin fragment 1.2 (F1.2), thrombin–antithrombin (TAT) complexes, thrombin (FIIa), APC, and D-dimer (DD) are expressed in relation to the respective upper limit of the reference ranges. *Green* , no increase; *yellow* , up to twofold-increase; *orange* , up to fivefold-increase; *red* , more than fivefold-increase; *gray* , not measured. The number of stars shown behind the type of surgery represents the number of RBCs given.

To follow changes in coagulation activity on an individualized basis, a color-coded scheme was applied ( Fig. 3 ). Data were stratified in relation to the upper limits of the reference ranges. Green indicates no increase and yellow indicates an up to twofold increase, while data colored in dark yellow or red showed a more than twice or more than fivefold increase, respectively.

At baseline, seven patients showed no increase of all five parameters. Five patients showed a moderate increase in thrombin formation as characterized by increased levels of at least one of the thrombin markers. In seven patients, an underlying thrombin activity was associated with increased APC levels, while D-dimer was within the reference range. Six patients showed moderately increased ( *n* = 5) or increased D-dimer levels without an increase in thrombin or APC. In four patients, thrombin and D-dimer levels but not APC plasma levels were increased, and in six patients, the biomarker pattern indicates increased thrombin formation associated with increased APC formation and increased D-dimer levels.

The activity patterns measured during surgeries indicate a strong increase in plasma levels of thrombin. This increase was not influenced by the activity status at baseline and was associated with increased TAT levels in the majority of patients, whereas F1.2 levels showed only a moderate increase. Among the 22 (63%) patients showing APC levels below the LLOQ at baseline, only 1 patient showed no increase in APC plasma levels despite a significant increase in thrombin and TAT levels. Among the nine (26%) patients showing moderately increased APC levels at baseline, seven patients showed a significant increase in APC levels during surgery, while APC levels in two patients remained stable. Stable APC levels throughout surgery were also observed in the four (11%) patients showing a more than fivefold APC increase at baseline.

Among the 19 patients showing D-dimer levels within the reference range at baseline, 10 showed increased D-dimer levels at end of surgery. The strongest increase in D-dimer was observed in the group of 16 patients showing increased levels at baseline. Twenty-four hours after end of surgery, 24 (69%) patients showed elevated D-dimer levels.

## Discussion

This study demonstrates enhanced but balanced systemic coagulation activity in response to localized coagulation activation as evidenced by increased levels of active thrombin and APC in the venous circulation of patients undergoing elective orthopaedic surgeries. To assess whether or not the size of the wounded area influences the rate of the systemic coagulation activation, patients undergoing major and minor surgeries were included into the study. The higher severity of surgical trauma in the major surgery group as stratified following ACH/AHA criteria was confirmed by significantly longer intervention times, increased rates of blood loss, and the differences in the need of red blood cell transfusions between both study groups. While one might expect that these differing conditions will yield divergent biomarker kinetics, no distinct patterns were observed between groups or in patients that received transfusions ( Fig. 3 ). However, while perioperative infusion volumes had not been monitored, significantly higher infusion volumes should have been applied within the major surgery group. Thus, it cannot be ruled out that, to some extent, the presented results are affected by dilution effects. To rule out that the kinetics of injury-related coagulation activation were influenced by critically low plasma levels of coagulation factors, only patients showing plasma levels of coagulation factors within the reference ranges were included into the study.

Fully active thrombin becomes detectable in the circulating blood immediately after start of surgery and remains increased during the time of surgery. This indicates that despite the high pressure of endogenous inhibiting mechanisms, the amount of thrombin that is released into the circulation is high enough to yield plasma levels of thrombin ranging between 10 and 100 pM. Although these concentrations are below the threshold values of 500 and 800 pM that are required to activate platelets and factors V and XIII, 3 one can expect that these plasma levels induce a hypercoagulable state when reaching low flow areas or areas of circulating flow such as in the valve pockets. 14 15

To estimate the relation between the total amount of thrombin formed and the amount of thrombin that is released into the circulating blood, plasma levels of F1.2 were compared with plasma levels of thrombin and TAT. Plasma levels of F1.2 significantly increased during surgery, whereas plasma levels of TAT were measured within the same molar ranges as F1.2. Considering the longer half-life of F1.2 of approximately 120 minutes as compared with 40 minutes of TAT, 5 significantly higher plasma levels of F1.2 should be expected. These data suggest that plasma levels of F1.2 underestimate the amount of thrombin actually formed. This finding supports previously published data. 16 One explanation for the difference between expected and measured F1.2 concentrations could be that parts of F1.2 remain bound to phospholipid surfaces within the growing clot and are not released into the circulation. Another explanation is that parts of F1.2 are altered by further proteolytic processing, whereas TAT represents a stable end product. 17 Regardless of the underlying mechanisms, these data demonstrate that F1.2 levels should be taken with caution when data on the amount of thrombin formation in response to localized coagulation activation are required. Hence, we were not able to assess the amount of thrombin that is formed at the wounded area. As expected, the increase in thrombin was associated with a rapid increase in plasma levels of APC, indicating that a substantial part of thrombin that is released from the wounded area into the circulation binds to TM on the endothelial cell surface. Plasma levels of APC showed a high degree of interindividual variability. This indicates that other factors besides thrombin modulate the APC formation capacity such as the density of the endothelial protein C receptor and TM on the endothelial cell surface. Moreover, plasma levels of APC are influenced by concentrations of APC inhibitors such as the protein C inhibitor and α-2-macroglobulin. However, one could speculate that a disbalance between the amount of thrombin formed and the subsequent APC response will induce a hypercoagulable state and thereby contributes to the increased risk of thrombosis in individual patients undergoing surgical interventions.

To study the kinetics of the surgery-related fibrinolytic response, we measured plasma levels of D-dimer. D-dimer is a composite measure for reactions downstream of thrombin generation including formation of cross-linked fibrin and its subsequent degradation by fibrin. Throughout the surgeries, D-dimer levels remained stable and significantly increased relative to baseline only at the end of surgeries in the group of major surgeries. This can be explained by the greater size of the wounded area in major surgeries resulting in higher concentrations of cross-linked fibrin. In both groups, D-dimer levels were found to be significantly increased 24 hours after end of surgery. This delay between increase in plasma levels of thrombin and APC on the one site and the increase in D-dimer on the other site indicates that the fibrinolytic response is substantially slower when compared with the fast pro- and anticoagulant responses.

Unexpectedly, the increase in all three thrombin markers showed no significant differences between the minor and major surgery groups. This indicates that the level of coagulation activation over time is comparably high in both groups. Most likely this can be explained by the surgical strategy that favors atraumatic techniques and restricts the tissue damage per time unit to a limited area even in major surgeries.

One might suspect that the rate of coagulation activation in response to the surgical trauma will be influenced by the activity levels at baseline. By analyzing the activity patterns on an individualized basis, a group of six patients was identified who showed increased coagulation activities at baseline as characterized by enhanced levels of thrombin markers, and/or APC and D-dimer. In this small subgroup, the rate of subsequent coagulation activation during surgery was comparable to the increase observed in patients showing no or only moderate increased basal coagulation activities. This demonstrates that the level of coagulation activity before start of surgery only marginally influence the coagulation activity that is achieved during surgery in patients without clinically relevant coagulation disorders. Factors that occur during surgery such as induction of TF expression in circulating monocytes might be more relevant. 18

To study whether changes in the systemic activity levels of coagulation occurring during surgery can be monitored by in vitro thrombin generation testing, we additionally performed CAT analysis. One might expect that the increase in systemic activity levels will shorten the lag time or will result in higher peak values. Overall, however, CAT parameters remained within reference ranges and unaltered relative to baseline throughout the surgeries. These results demonstrate that in vitro thrombin generation testing, applying a final TF concentration of 5 pM, cannot detect surgery-related changes in systemic coagulation reactions. This is supported by previously published data and is probably a consequence of rapid inactivation of thrombin and APC by endogenous inhibitors in the blood sampling tube rather than by the concentration of TF used. 19 In the OECA setting, reversible inhibitors of thrombin and APC are added to the sampling tube to block these inhibiting reactions.

Another potential limitation of the study might be that a peripheral venous access device was used for blood sampling during surgery, whereas blood samples before and after surgery were collected by puncturing an antecubital vein. While it seems unlikely that the different collection sites affect the results, it cannot be excluded that the biomaterial surface led to an artificial increase of coagulation markers although the flush then waste method was used during blood sampling. Moreover, the flush and waste method limits the number of blood sampling points due to the loss of blood.

The data presented here demonstrate that controlled surgical trauma induces a rapid change in systemic coagulation reactions as mainly characterized by increased levels of thrombin in the circulating blood. Maintenance of the hemostatic balance is achieved through rapid activation of the protein C pathway and inactivation of thrombin through stoichiometric inhibitors such as AT. The fibrinolytic response starts in the early postoperative period as indicated by a significant increase of D-dimer levels after end of surgery. There was no difference in the rate of systemic coagulation activation among the groups of patients undergoing major and minor surgeries. In general, activity pattern analysis could be a helpful tool to estimate the prothrombotic potential of surgical interventions on an individualized basis.

## References

[1] Monroe D M Hoffman M What does it take to make the perfect clot? Arterioscler Thromb Vasc Biol 2006 26 01 41 48 16254201 10.1161/01.ATV.0000193624.28251.83

[2] Grover S P Mackman N Tissue factor: An essential mediator of hemostasis and trigger of thrombosis Arterioscler Thromb Vasc Biol 2018 38 04 709 725 29437578 10.1161/ATVBAHA.117.309846

[3] Brummel K E Paradis S G Butenas S Mann K G Thrombin functions during tissue factor-induced blood coagulation Blood 2002 100 01 148 152 12070020 10.1182/blood.v100.1.148

[4] Huntington J A Serpin structure, function and dysfunction J Thromb Haemost 2011 9 01 26 34 21781239 10.1111/j.1538-7836.2011.04360.x

[5] Rühl H Berens C Winterhagen A Müller J Oldenburg J Pötzsch B Label-free kinetic studies of hemostasis-related biomarkers including d-dimer using autologous serum transfusion PLoS One 2015 10 12 e0145012 26658824 10.1371/journal.pone.0145012 PMC4684386

[6] Dahlbäck B Villoutreix B O The anticoagulant protein C pathway FEBS Lett 2005 579 15 3310 3316 15943976 10.1016/j.febslet.2005.03.001

[7] Rühl H Müller J Harbrecht U Thrombin inhibition profiles in healthy individuals and thrombophilic patients Thromb Haemost 2012 107 05 848 853 22274722 10.1160/TH11-10-0719

[8] Haynes L M Orfeo T Mann K G Everse S J Brummel-Ziedins K E Probing the dynamics of clot-bound thrombin at venous shear rates Biophys J 2017 112 08 1634 1644 28445754 10.1016/j.bpj.2017.03.002 PMC5406282

[9] Haynes L M Dubief Y C Orfeo T Mann K G Dilutional control of prothrombin activation at physiologically relevant shear rates Biophys J 2011 100 03 765 773 21281592 10.1016/j.bpj.2010.12.3720 PMC3030158

[10] Müller J Becher T Braunstein J Profiling of active thrombin in human blood by supramolecular complexes Angew Chem Int Ed Engl 2011 50 27 6075 6078 21591028 10.1002/anie.201007032

[11] Müller J Friedrich M Becher T Monitoring of plasma levels of activated protein C using a clinically applicable oligonucleotide-based enzyme capture assay J Thromb Haemost 2012 10 03 390 398 22236082 10.1111/j.1538-7836.2012.04623.x

[12] Fleisher L A Fleischmann K E Auerbach A D 2014 ACC/AHA guideline on perioperative cardiovascular evaluation and management of patients undergoing noncardiac surgery: executive summary: a report of the American College of Cardiology/American Heart Association Task Force on practice guidelines. Developed in collaboration with the American College of Surgeons, American Society of Anesthesiologists, American Society of Echocardiography, American Society of Nuclear Cardiology, Heart Rhythm Society, Society for Cardiovascular Angiography and Interventions, Society of Cardiovascular Anesthesiologists, and Society of Vascular Medicine Endorsed by the Society of Hospital Medicine J Nucl Cardiol 2015 22 01 162 215 25523415 10.1007/s12350-014-0025-z

[13] Hemker H C Giesen P Al Dieri R Calibrated automated thrombin generation measurement in clotting plasma Pathophysiol Haemost Thromb 2003 33 01 4 15 12853707 10.1159/000071636

[14] Dydek E V Chaikof E L Simulated thrombin responses in venous valves J Vasc Surg Venous Lymphat Disord 2016 4 03 329 335 27318053 10.1016/j.jvsv.2015.09.005 PMC4913030

[15] Elizondo P Fogelson A L A mathematical model of venous thrombosis initiation Biophys J 2016 111 12 2722 2734 28002748 10.1016/j.bpj.2016.10.030 PMC5192478

[16] Lee D Nayak S Martin S W Heatherington A C Vicini P Hua F A quantitative systems pharmacology model of blood coagulation network describes in vivo biomarker changes in non-bleeding subjects J Thromb Haemost 2016 14 12 2430 2445 27666750 10.1111/jth.13515

[17] Lippi G Cervellin G Franchini M Favaloro E J Biochemical markers for the diagnosis of venous thromboembolism: the past, present and future J Thromb Thrombolysis 2010 30 04 459 471 20213258 10.1007/s11239-010-0460-x

[18] Johnson G J Leis L A Bach R R Tissue factor activity of blood mononuclear cells is increased after total knee arthroplasty Thromb Haemost 2009 102 04 728 734 19806259 10.1160/TH09-04-0261

[19] Joly B Barbay V Borg J Y Le Cam-Duchez V Comparison of markers of coagulation activation and thrombin generation test in uncomplicated pregnancies Thromb Res 2013 132 03 386 391 23962423 10.1016/j.thromres.2013.07.022

