# Supplementary material for: Activity Pattern Analysis Indicates Increased but Balanced Systemic Coagulation Activity in Response to Surgical Trauma
Source: TH Open. 2018 Oct 1;2(4):e350–6. doi: 10.1055/s-0038-1673390 (PMC6524900; doi:10.1055/s-0038-1673390)
Supplement: Supplementary file 1 — Supplementary Material [file 10-1055-s-0038-1673390-s180036.pdf]

Supplementary Table 1 (A) Demographic and clinical data of the major surgery group

Sex	Age (y)	Type of surgery	OP time (min)	Blood loss (mL)	RBC transf. (units)
F	69	THP	94	350	2
M	40	THP	106	400	0
M	70	THP	108	250	0
F	70	THP	85	500	2
M	82	Spondylodesis	281	600	0
F	48	Nucleotomy	98	50	0
F	58	Spondylodesis	229	450	1
M	48	Nucleotomy	233	110	0
M	51	Nucleotomy	100	100	0
F	27	Spondylodesis	98	250	0
M	30	ORIF of radial head fracture	182	80	0
M	36	ACL reconstruction	100	0	0
M	65	Subtalar arthrodesis	324	150	1
F	26	ACL reconstruction	112	0	0
M	29	ACL reconstruction	130	0	0
M	34	ORIF of calcaneal fracture	180	200	0
M	65	Spondylodesis	425	1500	4

Abbreviations: ACL, anterior cruciate ligament; ORIF, open reduction internal fixation; THP, total hip arthroplasty

Supplementary Table 1 (B) Demographic and clinical data of the minor surgery group

Sex	Age (y)	Type of surgery	OP time (min)	Blood loss (mL)	RBC transf. (units)
F	58	Implant removal (hip)	74	150	0
M	62	Kyphoplasty	25	0	0
F	86	Kyphoplasty	32	0	0
M	28	WA and ORIF of distal radius fracture	124	50	0
F	24	Shoulder arthroscopy	60	0	0
F	77	Kyphoplasty	67	0	0
M	34	Implant removal lower leg	110	50	0
M	45	Implant removal wrist	100	0	0
M	37	Implant removal lower leg	90	0	0
M	35	Knee arthroscopy	36	0	0
M	57	ORIF of Weber fracture	27	0	0
M	58	Finger amputation	36	0	0
F	63	Kyphoplasty	23	0	0
F	64	Knee arthroscopy	52	0	0
F	47	Decompression	105	50	0
M	35	ORIF of clavicular fracture	61	0	0
F	62	Kyphoplasty	43	0	0
F	62	Kyphoplasty	19	0	0

Abbreviations: ACL, anterior cruciate ligament; ORIF, open reduction internal fixation; WA, wrist arthroscopy.

Supplementary Table 2 Determined biomarker plasma concentrations and correlation of data

		Time point																								
		Pre-op					Incision					Mid-op					End-op					Post-op				
		F1.2	TAT	FIIa	APC	DD	F1.2	TAT	FIIa	APC	DD	F1.2	TAT	FIIa	APC	DD	F1.2	TAT	FIIa	APC	DD	F1.2	TAT	FIIa	APC	DD
Type of surgery	Grade	pM	pM	pM	pM	µg/mL	pM	pM	pM	pM	µg/mL	pM	pM	pM	pM	µg/mL	pM	pM	pM	pM	µg/mL	pM	pM	pM	pM	µg/mL
Kyphoplasty	Minor	190	21.6	<0.46	<0.39	0.32	153	34.5	<1.06	<0.39	0.36	178	88.6	3.41	9.11	0.42	247	136.4	4.48	11.06	0.45	184	45.4	<0.46	<2.06	1.95
Knee arthroscopy	Minor	133	<21.3	<0.46	<0.39	0.26	115	<21.3	1.13	2.28	0.20	841	1086.1	8.82	4.95	1.18	353	314.8	286.10	<2.06	0.22	258	42.7	<0.46	<0.39	0.23
Knee arthroscopy	Minor	156	<21.3	<0.46	<0.39	0.30	118	<21.3	<1.06	2.18	0.33	376	433.3	10.44	<2.06	0.52	169	67.9	3.42	4.16	0.30	75	25.4	<1.06	<2.06	0.34
THP	Major	111	33.5	<0.46	<0.39	0.27	124	23.4	13.62	17.28	0.27	285	153.4	17.71	22.74	0.51	965	1769.2	149.05	33.93	2.14	118	68.6	<0.46	<0.39	0.89
Spondylodesis	Major	93	26.7	<0.46	<2.06	<0.17	94	41.9	<0.46	<2.06	<0.17	142	117.6	25.64	3.47	<0.17	134	70.5	<0.46	2.47	0.18	88	<21.3	<0.46	<2.06	0.28
Nucleotomy	Major	130	<21.3	<0.46	<0.39	0.29	133	25.4	2.63	<0.39	0.27	502	485.9	261.30	47.92	2.28	145	<21.3	4.41	<0.39	0.30	123	<21.3	3.86	<0.39	0.31
Posterior instrumentation	Major	95	24.4	<1.06	<2.06	0.35	366	352.4	5.66	3.53	1.93	256	373.5	5.54	57.34	0.95	922	722.6	8.50	12.76	1.69	225	45.0	1.36	<2.06	1.41
Nucleotomy	Major	158	28.0	2.23	<0.39	0.30	232	160.7	12.86	22.58	0.31	214	198.8	29.92	25.55	0.32	468	360.9	64.06	26.03	0.37	209	25.7	1.34	5.69	0.37
Decompression	Minor	124	78.2	<0.46	<2.06	0.25	114	26.3	29.10	4.96	0.24	229	199.8	76.51	33.88	0.28	272	242.6	69.43	8.52	0.27	146	21.7	<0.46	2.14	0.35
THP	Major	149	41.9	<0.46	<0.39	<0.17	101	27.6	<0.46	<2.06	<0.17	118	46.0	<0.46	<2.06	<0.17	420	393.1	10.64	37.36	5.37	182	80.1	<0.46	<2.06	0.40
Implant removal (hip)	Minor	187	57.5	1.47	<2.06	0.42	161	<21.3	<1.06	<2.06	0.40	281	204.6	32.95	6.64	1.02	499	276.8	39.71	18.29	0.71	193	28.8	1.36	<2.06	0.47
Finger amputation	Minor	405	53.4	<1.06	<2.06	0.34	2130	1057.-2	21.99	8.14	0.38	4110	2292.1	55.35	7.72	0.47	2910	1953.0	82.32	9.83	0.38	425	57.3	<0.46	<2.06	0.50
ACL reconstruction	Major	30	32.9	4.54	3.93	0.26	120	39.4	<1.06	5.46	0.28	140	68.7	9.49	2.72	0.27	160	46.4	4.67	3.97	0.27	689	61.6	<0.46	8.29	0.85
ACL reconstruction	Major	130	36.5	1.06	2.22	0.31	170	55.6	4.27	2.27	0.32	187	43.5	4.47	3.88	0.31	47	89.6	2.10	22.08	0.86	81	52.7	<0.46	3.15	0.52
Implant removal lower leg	Minor	165	75.9	<0.46	27.05	0.36	104	49.6	<1.06	16.25	0.35	116	78.6	23.62	20.59	0.36	128	88.2	142.12	21.33	0.39	170	53.8	<0.46	35.40	1.05
Kyphoplasty	Minor	171	70.7	6.68	3.13	0.19	151	25.4	8.01	<2.06	0.19	289	290.4	39.93	2.81	0.43		1979.7	286.10	3.22	0.89	130	29.0	2.46	<2.06	0.20
Implant removal wrist	Minor	139	49.8	11.94	13.01	0.18	147	86.6	<1.06	16.30	<0.17	220	194.0	4.80	11.35	<0.17	362	271.2	3.09	15.00	<0.17	260	105.4	<1.06	15.11	1.08
ACL reconstruction	Major	430	69.2	1.93	4.47	0.31	665	171.6	4.03	<2.06	0.29	1173	206.7	12.45	5.28	0.23	376	192.7	12.22	<2.06	0.24	383	43.7	<0.46	<2.06	1.37
Kyphoplasty	Minor	347	542.4	90.78	5.53	0.32	308	583.3	31.13	11.21	0.26	1051	2251.6	59.16	9.36	14.32	285	403.4	9.76	24.36	0.33	101	<21.3	<0.46	<0.39	0.20
Kyphoplasty	Minor	55	<21.3	<0.46	<2.06	0.62	88	28.5	<0.46	20.57	0.85	85	24.6	<0.46	20.16	0.75	90	26.3	<0.46	<2.06	0.70	96	<21.3	<0.46	2.30	0.76
Kyphoplasty	Minor	116	<21.3	<0.46	<0.39	0.57	186	64.8	21.93	31.80	0.51						550	493.9	25.83	34.85	1.61	204	26.2	<1.06	<0.39	1.18
WA & ORIF distal rad frac	Minor	171	31.9	<0.46	<2.06	1.72	94	33.5	<1.06	4.31	1.25	170	128.0	11.53	9.53	1.26	144	68.5	7.69	4.52	1.36	121	47.9	<1.06	20.50	1.12
ORIF of Weber fracture	Minor	127	35.0	<0.46	<2.06	0.68	155	31.9	1.73	2.19	0.86	266	123.3	41.30	3.65	1.03	346	163.6	13.06	3.31	1.13	180	34.5	<0.46	<2.06	0.75
THP	Major	254	<21.3	<0.46	<2.06	0.63	233	<21.3	3.13	<2.06	0.49	253	26.6	4.14	4.77	0.47	775	422.6	20.82	45.96	4.42	231	106.2	8.58	4.32	0.87
THP	Major	224	34.3	<0.46	<0.39	1.00	173	28.4	2.07	<0.39	0.66	235	70.9	6.59	<2.06	0.64	624	473.6	8.64	21.07	8.38	122	62.2	<0.46	<0.39	1.04
Nucleotomy	Major	150	23.4	3.90	<0.39	0.66	430	434.5	32.42	<0.39	0.63	1051	1344.4	51.24	<0.39	1.80	355	401.1	20.22	<0.39	0.72	146	21.9	1.50	<0.39	0.78
ORIF of radial head fracture	Major	105	30.3	2.31	<2.06	1.98	125	134.0	8.26	<2.06	1.69	277	243.2	8.15	6.32	1.85	276	335.9	12.60	<2.06	1.73	80	41.0	<1.06	3.05	1.65
Spondylodesis	Major	700	41.1	<0.46	<0.39	1.52	414	33.5	<0.46	<0.39	1.14	448	83.7	<0.46	<2.06	1.19	541	246.5	<0.46	9.32	2.15	519	50.6	<0.46	3.10	2.07
Shoulder arthroscopy	Minor	677	64.7	1.29	<2.06	1.43	167	36.3	3.91	2.44	0.31	239	78.4	5.41	6.62	0.32	196	58.0	1.63	15.53	0.75	618	38.3	<1.06	2.25	1.10

Supplementary Table 2 (*Continued*)

		Time point																								
		Pre-op					Incision					Mid-op					End-op					Post-op				
		F1.2	TAT	FIIa	APC	DD	F1.2	TAT	FIIa	APC	DD	F1.2	TAT	FIIa	APC	DD	F1.2	TAT	FIIa	APC	DD	F1.2	TAT	FIIa	APC	DD
Implant removal lower leg	Minor	165	33.1	<0.46	22.15	1.07	149	36.7	<0.46	19.73	1.01	210	147.6	3.97	38.41	1.11	263	103.2	4.99	25.30	0.25	142	57.3	<1.06	<0.39	1.58
Subtalar arthrodesis	Major	125	27.8	<1.06	7.05	0.68	230	101.0	11.22	3.83	1.28	593	400.0	69.44	6.62	1.03	328	209.2	259.62	58.20	3.91	216	37.0	<0.46	3.05	1.02
Kyphoplasty	Minor	222	41.1	<1.06	8.52	1.77	264	205.9	7.44	17.90	1.58	459	504.3	20.05	53.75	1.72	600	1047.6	23.16	61.85	2.81	239	97.0	3.02	<0.39	2.95
ORIF of clavicular fracture	Minor	211	119.1	26.88	19.00	0.89	268	139.6	5.31	17.71	0.89	626	686.6	69.84	43.43	3.10	831	599.6	58.95	13.19	0.92	563	100.8	<0.46	2.28	1.06
ORIF of calcaneal fracture	Major	87	47.6	<1.06	5.46	1.11	102	65.6	1.63	6.54	1.25	172	86.3	3.40	7.07	1.30	583	494.3	6.37	32.61	1.28	151	36.9	<0.46	<2.06	0.92
Spondylodesis	Major	193	48.1	1.36	3.21	0.60	215	49.9	<1.06	<2.06	0.56	338	60.8	<1.06	6.42	0.96	343	103.1	2.08	6.52	2.98	136	61.2	<0.46	<0.39	1.01

Note: Lower limit of quantification (LLOQ): F1.2: 20 pM; TAT: 21.3 pM; FIIa: 1.06 pM; APC: 2.06 pM; DD: 0.17 µg/mL. Lower limit of detection (LOD): FIIa: 0.46 pM; APC: 0.39 pM.
